# Clinical Efficacy and Safety Profile of Lofexidine Hydrochloride in Treating Opioid Withdrawal Symptoms: A Review of Literature

**DOI:** 10.7759/cureus.4827

**Published:** 2019-06-04

**Authors:** Saif Ur Rehman, Muhammad Haisum Maqsood, Hamza Bajwa, Asim Tameez Ud Din, Mustafa N Malik

**Affiliations:** 1 Internal Medicine, King Edward Medical University / Mayo Hospital, Lahore, PAK; 2 Internal Medicine, Rawalpindi Medical University, Rawalpindi, PAK; 3 Internal Medicine, District Headquarter Hospital, Rawalpindi, PAK

**Keywords:** opioid use disorder, lofexidine, clonidine, methadone, buprenorphine, diazepam, adverse effects, opioid withdrawal symptoms

## Abstract

Opioid use disorder (OUD) is a rapidly growing challenge worldwide and is characterized by an increase in dependence on opioids up to a point that a person loses control over the drug use. Multiple drugs are approved for its treatment, including methadone, buprenorphine, and diazepam. Although not approved, clonidine is also used for the treatment of OUD. On May 16, 2018, the United States Food and Drug Administration (FDA) approved a new drug lofexidine hydrochloride for the treatment of opioid withdrawal symptoms. Lofexidine is a centrally acting alpha two receptor agonist. It reduces the neurochemical surge by inhibiting the conversion of adenosine triphosphate (ATP) into cyclic adenosine monophosphate (cAMP) which in turn decrease the sympathetic outflow. This results in the improvement of withdrawal symptoms. When compared with methadone and buprenorphine, it is equally effective in controlling withdrawal symptoms. Its efficacy is also similar to clonidine with a better side effects profile. The adverse effects of lofexidine include bradycardia, hypotension, orthostasis, somnolence, sedation, dry mouth, and rebound elevations in blood pressure and prolongation of QT interval. Lofexidine is contraindicated in patients who are on beta-blockers and angiotensin converting enzyme inhibitors (ACE inhibitors). In our review, we have discussed the clinical efficacy and safety profile of lofexidine in treating opioid withdrawal symptoms and its comparison to other available treatment options.

## Introduction and background

Opioids are commonly used for the management of chronic pain. However, due to its long-term use, there has been an increase in the prevalence of opioid use disorder (OUD). It is estimated that around 32 to 36 million people are using opioids all around the world. Its illicit use is the major cause of human immunodeficiency virus (HIV) and hepatitis C spread and also causes deleterious psychological and social effects. Opioids are emerging as one of the leading causes of drug-associated deaths in North America. OUD is becoming a rapidly growing challenge and is characterized by increased dependency on opioids due to repeated and escalated doses so that the person has no longer control over the use of the drug. OUD also interferes with the daily life activities leading to social, psychological, and behavioral problems [1].

Opioid withdrawal symptoms manifest when a person abruptly stops taking the opioids. The onset and progression of these symptoms depend on the half-life of the opioid a person is taking. For instance, in case of short-acting opioids like oxycodone, symptoms start within 12 hours and taper off over four to seven days. In the case of long-acting opioids such as methadone, they can continue for a couple of weeks or more. The common symptoms mainly involve autonomic, including rhinorrhea, sneezing, coughing, lacrimation, sweating, and tremors. It can also include gastrointestinal symptoms like diarrhea, nausea, and vomiting which is attributed to the interaction with mu opioid receptors and psychiatric features such as insomnia, anxiety, and restlessness [2]. There are multiple scales available to assess the opioid withdrawal symptoms. The two commonly used are subjective opiate withdrawal scale (SOWS) and objective opiate withdrawal scale (OOWS). As implied by the name, in SOWS, the symptoms of opiate withdrawal are narrated by the patient. It includes 16 items that mainly comprise autonomic and gastrointestinal symptoms. OOWS involves a third person who rates the presence or absence of opiate withdrawal symptoms. A total of 13 items are included in this scale involving yawning, rhinorrhea, piloerection, sweating, lacrimation, mydriasis, tremors, restlessness, vomiting, muscle twitches, abdominal cramp, anxiety, and hot and cold flashes. All of these symptoms are scored as 1 point if it is present and 0 if it is absent. For yawning to be present the subject should have ≥1 episode and for rhinorrhea ≥3 sniffs in the period of observation. These scales are very useful for the evaluation of opiate withdrawal symptoms [3]

Currently, the Food and Drug Administration (FDA) approved drugs for the treatment of opioid use disorder (OUD) are methadone and buprenorphine. However, because of their potential for abuse and limited access, clonidine is often used to treat opiate withdrawal symptoms, although yet not approved by the FDA for this purpose. The use of clonidine is limited by sedation and hypotension caused by the doses required to treat OUD [4]. The United States FDA approved lofexidine hydrochloride to treat symptoms for opioid withdrawal on 16th May 2018, while in the United Kingdom, lofexidine has been approved since 1992 [5].

The primary aim of our study was to review the clinical efficacy and safety profile of lofexidine in treating opioid withdrawal symptoms. Our secondary aim was to compare lofexidine with other available management options, particularly clonidine.

## Review

Regardless of whether the opiate dependence has developed as a result of opiate prescription for chronic pain or for recreational use, the main issue for continued opioid abuse is to avoid the symptoms of withdrawal [6]. The first step in the management of opioid use disorder (OUD) is “stabilization”. In this step, the patients are shifted to another opioid drug (usually methadone or buprenorphine) so that they do not get the withdrawal symptoms but are also spared of the typical pleasurable effects of the opioids. The next step is the “detoxification” which can be described as clinically managed withdrawal. During this phase, the opioids are stopped and different drugs are used to prevent the symptoms of withdrawal from occurring. The third and final step is "rehabilitation", in which the focus is on prevention of relapse [7].

In the United States, it was found that there is a scarcity of the physicians and the facilities to manage OUD particularly in rural areas [8]. Lofexidine is a new agent for the management of opioid withdrawal symptoms. It is a structural analog of clonidine but has a higher affinity and specificity for alpha (α) two A subtype of the receptors, thereby reducing the sympathetic outflow. This makes it more efficacious and suitable for the treatment of opiate withdrawal [9]. The alpha (α) two A subtype receptor is a Gi protein-coupled receptor. On binding of an agonist, it reduces the conversion of adenosine triphosphate (ATP) into cyclic adenosine monophosphate (cAMP) and thereby reduces the phosphorylation cascade. This allows inhibition of the sympathetic outflow and allows the parasympathetic portion of the autonomic nervous system (ANS) to dominate [10]. Most of the alpha two adrenergic agonists, including lofexidine, also act on the I-1 imidazoline receptors in the brain and brainstem. Binding to this receptor also produces sympatholytic effects [11]. Locus coeruleus is the area in the pons that has been implicated in the pathophysiology of opiate withdrawal. With opiate intoxication, norepinephrine production is decreased in this area and during withdrawal, there is a rebound increase in norepinephrine production. Cells in locus coeruleus in opiate-dependent individuals show increased fos protein (a proto-oncogene) production. Lofexidine has been shown to decrease the c-fos messenger RNA (m-RNA) and fos protein production in these cells [12].

Lofexidine that is used clinically is composed of two isomers, dexlofexidine, and levlofexidine. The first study showing the differential effects of two enantiomers showed that levlofexidine is 20 times more potent antihypertensive than dexlofexidine. Levlofexidine was also found to be a more avid binder at alpha two adrenoceptors. It also binds more favorably to alpha one receptor than dexlofexidine. However, the binding ability of a drug to a receptor must not be taken as a measure of efficacy, rather a method must be devised to measure the intracellular effect produced by either enantiomer, for example, by measuring the intracellular cAMP levels. This will be a more reliable and accurate measure of the efficacy of the enantiomers [13].

Lofexidine is metabolized in the body by oxygen dealkylation and forms two glucuronide metabolites which are inactive. Almost 90% of the drug is excreted through kidneys, either as inactive metabolites or unchanged drug. The oral bioavailability of this drug is almost 72%. Hence, we can say that almost 28% of the drug is lost due to the first-pass metabolism in the liver and never reaches the bloodstream [14].

Lofexidine has been approved for the treatment of opiate withdrawal in the United Kingdom (UK) since 1992 but it was approved for this purpose in the United States (US) in 2018 [15]. Lofexidine is only approved for the treatment of the symptoms of opiate withdrawal and is not approved for medication-assisted maintenance treatment or long-term rehabilitation. Moreover, lofexidine does not treat all the withdrawal symptoms because patients taking lofexidine during withdrawal still complained of aches, insomnia, pain and disrupted sleep [16]. In one study in animals, it was seen that lofexidine can potentiate the intoxicating effects of cocaine, which suggests that it can only be used in opiate withdrawal and might be ineffective in the treatment of withdrawal from other agents. It may also increase the risk of future cocaine abuse in polydrug abusers [17].

Lofexidine has been compared with both placebos and active comparators in the treatment of OUD. Both lofexidine and clonidine are more effective in subsiding the withdrawal symptoms. In placebo-controlled trials, lofexidine group experienced lesser severity of withdrawal symptoms, better retention rates, and better completion rates. Moreover, lofexidine had a better safety profile than clonidine. Studies have shown that lofexidine is less likely to cause hypotension as compared to clonidine at doses required to treat the symptoms of opioid withdrawal. But lofexidine caused a measurable decrease in the blood pressure (BP) of the patients which was predictable based upon its mechanism of action [18]. In a systematic review involving ten studies, out of which nine studies compared agonist-antagonist combination mainly comprising alpha two agonists (clonidine or lofexidine) and opioid antagonist (naltrexone or naloxone) versus treatment with alpha-two agonist alone; the results were very heterogeneous. While the investigators concluded that both lofexidine and clonidine are effective in treating OUD, they could not say whether one was superior to the other in terms of efficacy or reducing the duration of withdrawal as results were not consistent among the studies [19]. In another trial, eight healthy opioid-dependent volunteers were stabilized on methadone. After stabilization, they were pre-treated with lofexidine, clonidine, and placebo. After this pre-treatment, naloxone was given to induce the withdrawal. It was found that both lofexidine and clonidine prevented the expected increase in the heart rate and blood pressure as compared to placebo. However, none of them reduced the subjective discomfort of opiate withdrawal induced by naloxone. Hence, it was concluded that lofexidine even if given in higher doses is well-tolerated but it cannot relieve all opioid withdrawal symptoms. It is suggested that lofexidine should be combined with some other drugs for its effective use in rapid withdrawal [20]. One advantage of lofexidine over clonidine is a higher therapeutic index of the former, which makes the outpatient management possible as well. Some sustained release formulations are also available which can maintain the plasma steady state for longer periods of time than conventional doses (starting at 0.2 to 3.2 mg/day of maximum dose) [20].

In a study comparing lofexidine with diazepam in the treatment of opioid withdrawal symptoms, it was found that during the 14-day withdrawal period, lofexidine group scored consistently lower on the objective opioid withdrawal score (OOWS) as compared to diazepam group. But there was no statistically significant difference in the OOWS score on day three and four of the withdrawal (peak of withdrawal). Therefore, it was concluded that lofexidine is at least as effective as diazepam in the treatment of opiate withdrawal [21].

In a randomized double-blind controlled trial that was carried out in prison, lofexidine was compared to decreasing doses of methadone. It was concluded that there is no significant difference between the two treatment groups in terms of the severity of withdrawal symptoms, heart rate, and blood pressure [22]. In another trial comparing lofexidine and methadone, favorable results for lofexidine have been obtained in terms of reducing the days to detoxification [10.2 days for lofexidine and naloxone, 6.7 days for lofexidine and placebo, and 3.9 days for methadone (*p* < 0.001)] [23]. A trial in 1996 compared lofexidine and methadone in the inpatient management of opioid withdrawal. There was no statistically significant difference between the patients of two groups who completed the trial. Although there were more serious symptoms in the lofexidine treatment group from days three to seven and day 10 which was the last day of treatment, there was no difference in the treatment completion rates between the two groups [24]. Hence, to conclude, lofexidine is similar to methadone in terms of treating withdrawal symptoms and can be used as an alternative agent with minimal differences in side-effect profile.

A randomized controlled trial comparing the efficacy of methadone/lofexidine and buprenorphine/naloxone on outpatient basis in opiate-dependent individuals in UK concluded that both these regimes were equally effective in treatment of opiate withdrawal i.e. there were no significant differences between the two groups in terms of positive urine samples, treatment retention rate or cravings for the opioid. However, there was an earlier peak of the withdrawal symptoms along with relatively severe withdrawal symptoms in the methadone/lofexidine group [25]. In another trial, it was found that the effects of methadone/lofexidine and buprenorphine/naloxone were comparable, and no significant efficacy difference was found. Although the patients in the methadone/lofexidine group showed better retention and a lesser amount of opiate-positive urine cultures than buprenorphine/naloxone group in the maintenance phase. In the induction/stabilization phase, the severity of withdrawal symptoms and cravings were significantly lower in the methadone/lofexidine group. However, in the detoxification phase, withdrawal symptoms were relatively severe, and the peak of withdrawal occurred relatively earlier for methadone/lofexidine group [25]. 

In Asian countries, because of the “zero tolerance” drug use policy, the standard treatment (buprenorphine/methadone) is not available for the treatment of opioid withdrawal. The standard pharmacological regimen used in these (Asian) countries is oral diazepam for seven to ten days. Diazepam, because of its muscle relaxant and anxiolytic properties, alleviates some of the symptoms associated with opioid withdrawal. But most of the patients who are abusing opioids are also concomitantly abusing benzodiazepines. Furthermore, because of their abuse potential, the use of benzodiazepines is relatively contraindicated in patients with a history of substance abuse [26]. During the opioid withdrawal, day three and four of the withdrawal are reported as the peak withdrawal and the withdrawal symptoms during these days are most severe [24]. In a study comparing diazepam and lofexidine for the treatment of opioid withdrawal in which the primary outcome was objective opiate withdrawal score (OOWS) on day three and four, there was no significant difference in OOWS in the two groups but the scores were consistently lower for lofexidine group since day two. One of the secondary outcome measures in this study was the pupil size during the treatment. There was no statistically significant difference in pupil size between two groups, but the size was consistently smaller for lofexidine group up until day 12. In terms of treatment retention, on day four of the study (peak withdrawal) number of patients who self-discharged was double in the diazepam group than in the lofexidine group. However, at the end of the study, there was no significant difference in the retention rates in the two groups. The most common adverse events encountered in the lofexidine group were hypotension, bradycardia, and dry mouth. Three out of /108 participants experienced numbness (hands, feet, and tongue). This numbness was a new adverse event recorded in this study but further studies are required to probe its association with lofexidine. This study concluded that lofexidine is non-inferior to diazepam and is associated with better treatment retention in the treatment of opioid withdrawal. The number of participants who self-discharged on day four was double in the diazepam group as compared to lofexidine group. This is of importance because withdrawal symptoms are supposed to be most severe during this time. It can be concluded that lofexidine is associated with better retention rates as compared to diazepam in the treatment of opiate withdrawal [21].

Bradycardia hypotension, orthostasis, dizziness, somnolence, sedation, dry mouth, and rebound elevations in blood pressure occurred more frequently in the lofexidine group than the placebo group (Table 1). Lofexidine causes less hypotension than clonidine [19, 27]. Lofexidine can cause prolongation of QT interval and regular electrocardiography (ECG) monitoring is recommended in patients with liver, kidney or heart failure. Co-administration of methadone and lofexidine can also lead to a significant prolongation of QT interval [28]. Lofexidine has been found to be associated with decreased cognitive efficiency and higher doses have been seen to be associated with decreased ability to solve a mathematical problem [29]. Lofexidine should not be taken with other drugs that can decrease the heart rate or blood pressure, like beta blockers or angiotensin-converting enzyme inhibitors (ACE) inhibitors. Lofexidine is metabolized by cytochrome P450 2D6 CYP2D6. Drugs that inhibit this enzyme can lead to an increase in the blood levels of lofexidine. Based on its mechanism of action, it can be speculated that overdose of lofexidine will lead to similar symptoms as that of clonidine but the reports on lofexidine toxicity are lacking. Although there is no strong evidence, in order to treat lofexidine toxicity, we can use intravenous (IV) naloxone to produce a state of opioid withdrawal in opioid-dependent patients which can counteract some of the symptoms of lofexidine toxicity [30].

**Table 1:** Adverse effects of various drugs used in the treatment of opioid withdrawal symptoms

DRUGS	ADVERSE EFFECTS	REF.
Lofexidine	bradycardia, hypotension, orthostasis, dizziness, somnolence, sedation, dry mouth	[27]
Clonidine	sedation, dry mouth, hypotension, drowsiness, dizziness	[18, 31]
Buprenorphine	headache, nausea, constipation, vomiting	[31, 32]
Methadone	QT interval prolongation, constipation, nausea, sedation, respiratory depression, pruritus	[33]
Diazepam	drop in blood pressure, sleepiness, slow heart rate	[21]

## Conclusions

Lofexidine is a better non-opiate drug than clonidine for the treatment of opiate withdrawal symptoms because its efficacy is comparable to clonidine and it causes less severe hypotension and dizziness than clonidine. However, it is more expensive as compared to clonidine. Lofexidine is also found to be equally effective as buprenorphine/naloxone and methadone. When compared to diazepam, lofexidine is non-inferior to it and is associated with better treatment retention. In Asian countries, due to non-availability of standard treatment, diazepam is commonly used for opioid withdrawal symptoms. Hence, based on our review, it can be concluded that lofexidine is a good non-opioid alternative for treating opioid withdrawal symptoms with less adverse effects as compared to the standard therapies available.
